# Gene set bagging for estimating the probability a statistically significant result will replicate

**DOI:** 10.1186/1471-2105-14-360

**Published:** 2013-12-12

**Authors:** Andrew E Jaffe, John D Storey, Hongkai Ji, Jeffrey T Leek

**Affiliations:** 1 Department of Biostatistics, Johns Hopkins Bloomberg School of Public Health, Baltimore MD 21205, USA; 2 Lieber Institute for Brain Development, Johns Hopkins Medical Campus, Baltimore MD 21205, USA; 3 Lewis-Sigler Institute and Department of Molecular Biology, Princeton University, Princeton, NJ 08544, USA

**Keywords:** Gene set enrichment analysis, Gene expression, DNA methylation, Gene ontology

## Abstract

**Background:**

Significance analysis plays a major role in identifying and ranking genes, transcription factor binding sites, DNA methylation regions, and other high-throughput features associated with illness. We propose a new approach, called *gene set bagging*, for measuring the probability that a gene set replicates in future studies. Gene set bagging involves resampling the original high-throughput data, performing gene-set analysis on the resampled data, and confirming that biological categories replicate in the bagged samples.

**Results:**

Using both simulated and publicly-available genomics data, we demonstrate that significant categories in a gene set enrichment analysis may be unstable when subjected to resampling. We show our method estimates the replication probability (*R*), the probability that a gene set will replicate as a significant result in future studies, and show in simulations that this method reflects replication better than each set’s p-value.

**Conclusions:**

Our results suggest that gene lists based on p-values are not necessarily stable, and therefore additional steps like gene set bagging may improve biological inference on gene sets.

## Background

The gene expression program of cells can be organized into a diverse set of pathways that perform specific functions [1]. Human health depends on the functionality of these pathways; de-regulation at the pathway level may be more important for diseases like cancer than de-regulation of specific genes [2]. The most common statistical approach for identifying pathways of interest in a high-throughput experiment is to perform a significance analysis gene-by-gene and then summarize the significant hits using gene set or gene pathway analyses. Each pathway or gene-set analysis is performed once on the entire data set. However, there is variability in the identified gene sets due to both the instability in gene rankings from the original gene ranking analysis and from the pathway/set analysis. Furthermore, scientists are often interested in whether these results will replicate as significant in future similarly-designed studies using independent samples.

Here we propose a new approach to evaluate the stability of biological inference drawn from an experiment, and estimate the probability that the result replicates in future studies. Our approach, called *gene set bagging*, performs a resampling of the entire discovery algorithm - significance analysis and gene set enrichment - to identify the most stable and reproducible enriched gene sets. Bagging, also known as bootstrap aggregating, is traditionally used for assessing the predictive accuracy and stability of prediction models [3]. While bagging and bootstrapping procedures have been used for differential expression analyses [4] and other genome-wide applications [5-8] here we introduce a new bagging procedure for significance analysis. This procedure can be useful for both evaluating significance rankings and also for describing the most reproducible genes and biological gene sets within genomics experiments in a platform-independent fashion.

We perform resampling by drawing observations with replacement from the (full) original data set with sample size equal to the original, performing a significance analysis followed by gene set analysis, and then identifying which sets are enriched. We can identify which observed gene sets are consistently enriched in resampled data, and compute the gene set replication probability (*R*), a measure of gene set stability based directly on the biological quantity of interest, representing the probability that an observed gene set will be enriched in future experiments.

The replication probability (*R*) has some important advantages over the traditionally-reported p-value for summarizing gene set enrichment. The structure of the gene set testing problem is fundamentally different than other multiple hypothesis testing problems - correlations between genes, different gene set sizes, and different levels and fraction of differential expression within gene sets make the hypotheses fundamentally not comparable with standard significance testing [9,10]. We propose to estimate directly the probability that a gene set will replicate because an estimate of the probability of replication may be of more interest than a measure of statistical significance. Given the emphasis on replication in genetics/genomics studies, this replication probability may be another metric for directing molecular validation of important biological processes involved in human disease.

We perform our gene set bagging method on two types of genomics measurements: gene expression and DNA methylation. Even after adjusting the genomic data for potential batch effects, we demonstrate that some significant gene sets fail to replicate well, yet other non-significant sets have high replication rates. The results for these different genomic technologies suggest that the signal and noise structure of the specific genomic data type contribute greatly to stability of gene sets. We use a simulation study to assess replication across two simulated datasets, and evaluate the concordance between replication probability (*R*) and the traditionally-reported significance metric (P-value). In simulations we show that the replication probability better quantifies the chance that a significant gene set will be consistent across studies. Our results suggest that: (1) gene set enrichment analyses based on significance analysis may be unstable in some cases, and (2) gene set bagging is a resampling approach for measuring the stability of gene sets and estimating replicability of biological conclusions.

## Methods

For a given gene set, the goal is to estimate:

$$R_{l}=\text{Pr}(\text{Gene}\;\text{set}\;l\;\text{will be significant in a new study}).$$

The quantity *R*_*l*_ is useful as a measure of the stability of the significance of an identified gene set. Gene sets are frequently used to interpret the biological results of studies, so it is important to know if the biological interpretation would change if the study was repeated. This is particularly true since gene set analysis is subject to errors in annotation, variation due to technological noise, and variation due to biological noise. We define “replicability” as the ability to achieve similar results when experiments are rerun, and note this differs from “reproducibility”, which we view as the ability to run the analysis code again and get the same answer within a dataset [11].

As an example of our general approach, we focus on a real dataset examining the role of cigarette smoking on gene expression (further explained in the following “Datasets and implementation” section), which examined expression differences associated with smoking exposure in 40 smokers and 39 never-smokers. We define gene expression measurements *m*_*ij*_ for each of *j*=1,…,79 samples over *i*=1,…,*M* genes/probes (corresponding to gene *g*_*i*_) and a covariate of interest per sample (*z*_*j*_∈[ *currentsmoker*,*neversmoker*]). We first want to identify differentially expressed genes between the two outcome groups, so we calculate an empirical Bayes regularized t-statistic and resulting p-value for each gene [12]. We can convert these p-values to q-values to identify which genes are significant according to the false discovery rate. We then test for enrichment among the significant genes in *L* predefined gene sets using the usual hypergeometric test. Each gene set yields a p-value (*p*_*l*_,*l*=1,…,*L*), reflecting the degree of enrichment. Another approach to obtaining the gene set p-value (*p*_*l*_) is to calculate them directly from the significance ranks of the genes (thereby bypassing the need to call a particular set of genes significant). For example, the Wilcoxon rank-based gene set enrichment test [13] available in the limma Bioconductor package [14] can be used to test for a difference in the significance ranks of the genes in the gene set versus all of the other genes.

We then perform the gene set bagging algorithm over *B*=100 iterations. In each iteration, *b*, we resample the gene expression vectors of the 40 smokers and 39 never-smokers, respectively, with replacement. Each gene or probe yields a p-value via calculating a t-statistic in the resampled data, and these statistics are passed to a gene set analysis algorithm to produce a enrichment p-values for the gene sets ($p_{l}^{b}$, *l*=1,..,*L*), which are stored in column *b* of a *L*×*B* matrix, for *b*=1,2,…,*B*. For each row, which represents a gene set, we count the number of times each subsampled p-value ($p_{l}^{b}$) is less than *α* (here, 0.05), and divide it by the number of iterations (*B*), resulting in an estimate of the replication probability for that gene set (${\hat{R}}_{l}$).

Estimated replication probabilities ($\hat{R}$) are between 0 and 1, where 0 means that the gene set always had a p-value greater than *α* in every iteration, and 1 means that the category always had a p-value less than *α* in each iteration. For analyses where the gene ranking is stable and the gene set calculation is stable, the replication probability will be higher. This estimate of replication assesses the stability of the gene sets, and might be a better estimate of biological reproducibility than the traditionally reported p-values. Our goal is to identify the stable gene sets, akin to Meinshausen and Bühlmann (2010) [15] in selecting a more stable set of covariates in a regression model.

**Algorithm 1 Gene set bagging procedure:**
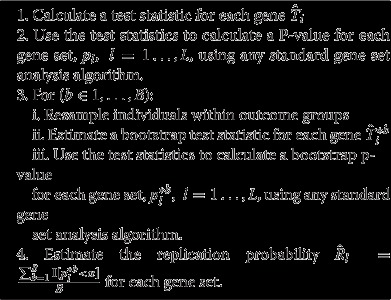


### Datasets and implementation

#### Simulated data

We designed two simulation studies to assess different properties of the replication probability based on the Affymetrix Human Genome 133 Plus 2.0 gene expression microarray. Basing the simulation on an existing array design, with probes annotated to genes that were already mapped to gene ontology categories, allowed us to realistically add differential expression signal to specific gene sets. We first selected a random sample of 100 gene sets to use in our simulation, which corresponded to 2288 unique genes. Then, for each simulation, we simulated genes via the following model:

$$m_{\text{ij}}=\beta_{0}+\beta_{i}z_{j}+\varepsilon_{\text{ij}}$$

where *ε*_*ij*_∼*N*(6,1), *β*_*i*_∼*N*(1,0.5) if *g*_*i*_ is differentially expressed, and *β*_*i*_=0 if *g*_*i*_ is not differentially expressed. The variables *m*_*ij*_ and *z*_*j*_ (defined above) correspond to the expression value and group label, respectively.

In Simulation 1, we generated 1000 datasets, where each consisted of 100 individuals (50 cases and 50 controls). For each dataset, we made 100 genes differentially expressed and computed the observed p-value (*p*_*l*_) and then the replication probabilities (${\hat{R}}_{l}$) for each gene set *l*=1,…,*L*. In Simulation 2, to directly assess the replication probability across two datasets with the same differentially expressed genes, we generated 100 pairs of datasets, where each dataset contained 50 individuals (25 cases and 25 controls). For each data set, we set 500 genes to be differentially expressed, with the same parameter settings from the above model. This simulation mimics a perfect replication of the gene-set experiment where all parameters are the same. On each dataset, we then computed observed p-values (*p*_*l*_) and replication probabilities (${\hat{R}}_{l}$) for each gene set *l*.

#### Gene expression: cigarette smoking data

We tested the gene set bagging method in a differential expression analysis with publicly-available data obtained from Gene Expression Omnibus (GSE17913). This study (initially approved by the Weill Cornell Medical College Institutional Review Board) examined the association of cigarette smoking with the oral epithelial transcriptome by comparing buccal biopsies in 39 never-smokers with 40 active-smokers using the Affymetrix Human Genome U133 Plus 2.0 microarray [16]. We processed the raw CEL files using the RMA algorithm to perform intra-array normalization and then performed quantile normalization to adjust for between-array biases [17].

We performed surrogate variable analysis (SVA) to adjust for potential batch effects [18,19]. Briefly, this approach identifies the number of right singular vectors that are associated with more variation than expected by chance, and then in the subsets of genes driving this variation, constructs a ‘surrogate’ variable for each subset. These surrogate variables are then included as covariates in our differential expression analysis (so that the model becomes: *m*_*ij*_=*β*_0_+*β*_*i*_*z*_*j*_+*γ*_*i*_S*V*_*j*_+*ε*_*ij*_).

We identified differentially expressed genes comparing cases and controls while controlling for the surrogate variables using an empirical Bayes approach [20]. To determine statistical significance, resulting p-values were converted to q-values to control for the false discovery rate [21] and all transcripts with q-values less than 0.05 were considered significant. We performed a full gene ontology analysis, and then ran the gene set bagging algorithm.

#### DNA methylation: brain tissue

This approach is likely generalizable to most genomics platforms, and we first tested this hypothesis using DNA methylation data processed on the Illumina HumanMethylation27 platform (obtained from GEO [GSE15745]) from a recent paper [22] that assessed quantitative trait loci using methylation and expression data in four different brain tissues (exempt from human subjects research due to being postmortem tissue from brain banks). Previous work has identified that DNA methylation signatures can distinguish brain tissues, and might play a role in determining and stabilizing normal brain differentiation [23]. We conducted our gene set bagging algorithm on the differential DNA methylation analysis between the frontal and temporal cortices. We performed the full differential methylation analysis comparing 131 front cortex and 126 temporal cortex samples using SVA and the empirical Bayes approach as described above. All probes with q-values less than 0.05 were considered significant. We performed a full gene ontology analysis on the gene associated with each probe (from the annotation table), and ran the gene set bagging algorithm.

## Results

### *R* estimates the probability a gene set will be significant in a repeated study

**Figure 1:**
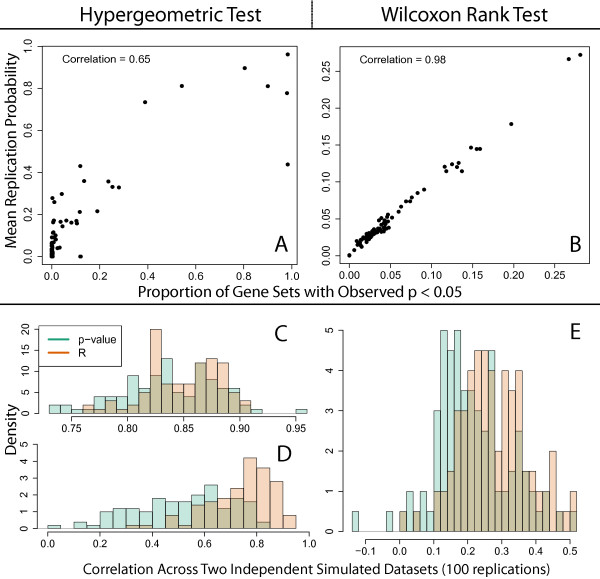
**Replicability assessed from the simulations.Simulation 1.** Observed gene set p-values based on the **(A)** hypergeometric and **(B)** Wilcoxon Rank tests and then subsequent replication probabilities were calculated. The x-axis is the proportion of observed p-values that are less than 0.05 for each gene set and the y-axis is the average replication probability for that gene set. Spearman correlations were calculated to avoid issues with non-linearity. **Simulation 2.** The gene set p-values *p*_*l*_ and replication probabilities ${\hat{R}}_{l}$ were calculated for each data set, where 100 pairs of data sets with common differentially expressed genes were simulated. The Spearman correlation of the gene set p-values *p*_*l*_, *l*=1,…,*L* was calculated for each pair of datasets, and analogously for the replication probabilities ${\hat{R}}_{l}$. The 100 resulting correlations of gene set p-values or replication probabilities for **(C)** all gene sets and **(D)** those significant in either paired dataset at *p*<0.05. The replication probability offers better correlation between independent datasets for significant gene sets, but similar correlation across all significant and non-significant gene sets, than the p-value for the hypergeometric test.

The interpretation of the replication probability reflects the underlying stability of each outcome group. We simulated 1,000 datasets from a common model (as described in section “Datasets and implementation”, Simulation 1), each with 100 differentially expressed genes. We then performed gene set analysis (based on gene sets described in section “Datasets and implementation”) using both the hypergeometric and Wilcoxon tests and calculated the replication probability estimates for each of gene set in each of the 1,000 simulated studies. The average replication probability estimate across all 1,000 repeated studies very closely approximates the frequency that a gene set is observed to be significant in those 1,000 studies (Figure 1A and 1B). In other words, the estimate of the replication probability is close to the probability a gene set will be significant in a repeated study.

### *R* correlates better with replication in repeated studies

Besides identifying which gene sets are the most stable, we can also assess how well the replication probability ($\hat{R}$) reflects biological replication by generating two independent simulated datasets with the same differentially expressed genes, meant to represent repeated studies of the same biological effect (described fully in section “Datasets and implementation”, Simulation 2). We performed traditional gene ontology analysis on both datasets, obtaining p-values for each gene set calculated from the hypergeometric distribution, and then performed our gene set bagging algorithm. There was very strong Spearman correlation between pairs of datasets across 100 simulation runs when all gene sets were considered regardless of whether the replication probability (median = 0.854, IQR: 0.826-0.876) or p-value (median = 0.836, IQR: 0.809-0.869) was used (Figure 1C). However, when only gene sets where at least 1 of 2 datasets was significant at *p*<0.05 per simulation run, the replication probability had much stronger correlation (median = 0.755, IQR = 0.678-0.817) than the p-value (median = 0.535, IQR: 0.387 - 0.648) (Figure 1D).

These results suggest that globally, there might not be a large difference between the replication probability and the p-value, but when there is any signal in a particular gene set, the replication probability better captures independent replication of that set in future studies. We also performed the more robust Wilcoxon rank rest on these simulated paired datasets, which also had less correlation between the resulting gene set p-values than the replication probability (Figure 1E). There were many fewer significant gene sets by this enrichment approach than the hypergeometric test, and it was rare that both independent datasets within a simulation were significant at *p*<0.05.

### *R* may add biological interpretability

**Figure 2:**
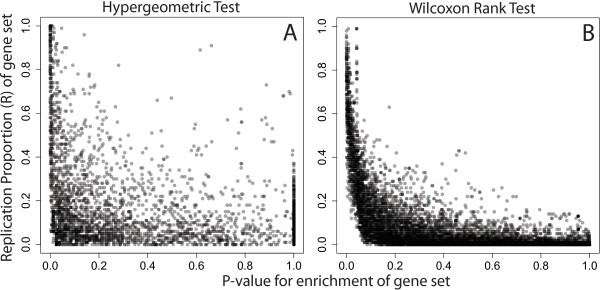
**Expression dataset gene set analysis, smokers versus never-smokers.** Gene set analyses were performed by the **(A)** hypergeometric and **(B)** Wilcoxon rank tests using gene sets defined by the Gene Ontology, and the replication of each gene set was assessed via our gene set bagging procedure (each point is one gene set). The relationship between the estimated replication probability ($\hat{R}$) and traditionally reported p-value appears much more concordant using the Wilxocon rank test.

While many gene sets have both small p-values and high replication probabilities, examining discordant gene sets may improve the biological interpretation of the research question at hand. For example, in the gene expression dataset (Figure 2), there were 8 GO categories with p > 0.05 and $\hat{R}>0.8$ under the hypergeometric test, including sets associated with phosphorylation (GO:0006468, GO:0016310), a process affected by cigarette smoking [24] and regulation of metabolic processes (GO:0019222, GO:0044267).

**Figure 3:**
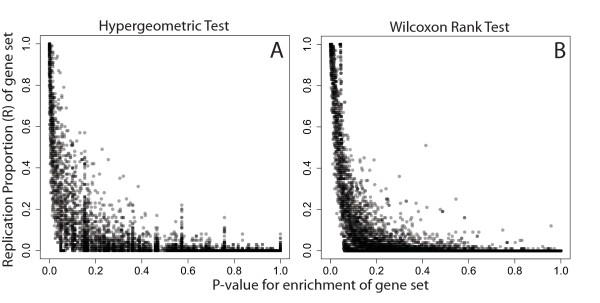
**DNA methylation dataset gene set analysis, human brain regional differences.** Gene set analyses and gene set bagging were performed by the **(A)** hypergeometric and **(B)** Wilcoxon rank tests using gene sets defined by the Gene Ontology. The relationship between the estimated replication probability ($\hat{R}$) and traditionally reported gene set p-value are only slightly more concordant with the Wilxocon rank test.

Similarly, examining the categories associated with DNA methylation differences across brain tissue types that had at least moderate replication and non-significant p-values demonstrates support for the gene set bagging approach as well as the shortcomings of relying on strict gene set p-value cutoffs for gene ontology analysis (Figure 3). Several biologically plausible GO categories for a comparison of methylation differences in brain tissues fell into the “marginally significant” bin of observed p-values between 0.05 and 0.1 but had consistent replication.

There were many smaller gene sets that had statistically significant p-values (p < 0.05) but never appeared in any of the resampled datasets ($\hat{R}=0$) in both the gene expression (32 gene sets) and DNA methylation datasets (12 gene sets). These represent very unstable gene sets, and should be interpreted with caution. Categories with ($p>0.05,\hat{R}>0.8$) would have been ignored in a traditional gene set analysis given their statistical significance measure, but might be biologically important to the question of interest. Likewise, gene sets with ($p<0.05,\hat{R}=0$) may be less biologically meaningful even though they are “statistically significant”.

We can characterize some global properties of the replication probability via these two datasets. Overall, in the cigarette smoking gene expression dataset, the correlation between the replication probability $\hat{R}$ is correlated with the number of significant genes in a gene set (*ρ*_*spearman*_=0.630). The gene set p-value shows a stronger correlation with the number of significant genes in a gene set (*ρ*_*spearman*_=−0.985). Both quantities are also correlated with the total number of genes in a gene set $\hat{R}$ (*ρ*_*spearman*_=0.569) and gene set p-value (*ρ*_*spearman*_=−0.544). We also observe that larger datasets lead to better estimates of replication via the replication probability. Comparing the smoking expression dataset (*N*=79) to the brain DNA methylation dataset (*N*=257), we note that the smaller study has more gene sets with $\hat{R}>0$ (7,373 versus 3,708) and has more gene sets with $0<\hat{R}\leq 0.15$. However, the larger dataset has more gene sets with $\hat{R}>0.15$.

### Relationship to the problem of regions

The set of test statistics corresponding to genes within an individual set can be viewed as a multivariate random vector. When viewed in this way, a gene set is significant if the vector of test statistics falls into a multi-dimensional region defined by the significance threshold. The replication probability is then a first-order approximation estimate of the posterior probability a gene set will be significant, assuming a non-informative prior distribution on the vector of test statistics. This problem has been considered in the case of multivariate normal data [25] and for estimating confidence in inferred phylogenies [26]. As has been previously pointed out, this posterior probability is a reasonable first approximation to the posterior probability in question, but should not be interpreted as a frequentist measure of statistical significance [25,27].

As an example of the relationship between the bootstrap and a posterior probability, suppose *z*_1_,…,*z*_*n*_∼*N*(*μ*,*σ*^2^). A non-informative prior distribution for the parameters (*μ*,*σ*^2^) is the Jeffrey’s prior [28]. The Jeffrey’s prior for *μ* is an improper uniform prior across the real line and the Jeffrey’s prior for $\sigma^{2}\propto \frac{1}{\sigma^{2}}$. Using these prior distributions, the posterior distribution for *μ* is $N(\bar{z},\tau^{2})$ where *τ*∼*InverseWishart*_*n*−1_((*ns*^2^)^−1^) and $s^{2}=\frac{1}{n}\overset{n}{\underset{i=1}{\sum }}{\left(z_{i}-\bar{z}\right)}^{2}$. In this case, since *μ* is one dimensional, the *InverseWishart* distribution is equivalent to an *InverseGamma* distribution. Drawing bootstrap samples from the *z*_*i*_ and recalculating the mean approximates sampling from the posterior distribution of *μ* (see supplemental R code). It is important to note that the variance of the posterior for *μ* is inflated compared to *σ*^2^ assuming a frequentist model [25,27]. Note that the p-values from these bootstrap samples should not be interpreted as measures of statistical significance, because they are no longer distributed uniformly.

## Discussion and conclusions

We have developed a resampling-based strategy for assessing the stability of gene sets which also estimates the probability a gene set will replicate (being statistically significant) in a future study. This direct approach to estimating replicability may be more useful than statistical significance for investigators who aim to identify stable and reproducible biological interpretations of their results. By utilizing resamplings of the observed data that respect the study design, the reproducibility of gene sets can be quantified, represented by the replication probability *R* of each gene set category across all subsamples. This approach can offer an additional metric beyond the gene set p-value for identifying important biological pathways. We have applied this method to gene expression and DNA methylation under two commonly-used enrichment metrics: the hypergeometric test and the Wilcoxon rank test. We demonstrated that some seemingly statistically significant GO categories fail to replicate consistently. A strength of our approach is the likely generalizability of this algorithm to other genomics applications, including incorporating bias-correcting approaches like SVA into the analysis, to assess the stability and replicability of significance results.

Gene sets with high replication probabilities and low p-values represent statistically significant, stable, and consistent sets that might best represent the underlying biology within the experiment. Overall, the Wilcoxon rank test appears more stable than the hypergeometric p-value, using simulated and real data. There was less disagreement between gene set p-values and replication probabilities, and the quantitative relationship between the replication probability and p-value was more precisely defined (Figure 1B and 2B). Given that most genomics studies require some form of external replication and that *R* appears more correlated with replication in future studies than p-values alone, we might also suggest following up gene sets that have high replication probabilities (*R*) even if the p-values are marginally, or even non-significant. The gene set bagging algorithm has been implemented in the R package “GeneSetBagging”, available through GitHub (https://github.com/andrewejaffe/GeneSetBagging). Users may choose different gene set p-value and replication probabilitiy cutoffs depending on their resources for follow-up studies.

Genomics studies often involve drawing the majority of biological conclusions from the results of a gene set analysis without assessing the stability of the results. We envision replication probabilities used in conjunction with standard measures of statistical significance, as the emphasis on replication in genetics and genomics makes the replication probability a useful quantity to estimate and use in conjunction with p-values. We have demonstrated that gene lists are not necessarily stable, and therefore additional steps like gene set bagging should be undertaken to improve the biological inference of a given study.

## Competing interests

The authors declare that they have no competing interests.

## Authors’ contributions

JTL and JDS designed the study, AEJ performed the analyses, AEJ HJ JDS and JTL wrote the manuscript. All authors read and approved the final manuscript.
